# Datasets on the nutritional and environmental (including biodiversity) characteristics of food products consumed in France

**DOI:** 10.1016/j.dib.2023.109518

**Published:** 2023-08-28

**Authors:** Samuel Le Féon, Florent Vieux, Christophe Geneste, Rozenn Gazan, Nicole Darmon, Jean-Louis Peyraud, Marion Tharrey, Joel Aubin

**Affiliations:** a UMR SAS^1^, INRAE, Institut Agro, 65 rue de Saint Brieuc, Rennes 35042, France; b MS-Nutrition, Marseille 13005, France; c Université de Montpellier, CIRAD, CIHEAM-IAMM, INRAE, Institut Agro, MOISA, Montpellier 34060, France; d INRAE, Institut Agro, PEGASE, Le Clos, Saint-Gilles 35590, France

**Keywords:** Life cycle assessment, Life cycle inventory, Biodiversity, Nutrition, Diet, Protein

## Abstract

Analysing the nutritional and environmental impacts of our current diets and promoting sustainable dietary shifts require quantified data on the characteristics of foods. We have jointly studied environmental and nutritional performances of more than 200 generic foods consumed in France, by combining and completing different databases. Several environmental issues calculated by Life Cycle Assessment (LCA) were selected, including impacts on biodiversity. This required to (1) model diets for given subpopulations; (2) adapt the LCA database of food products, Agribalyse 3.0, to link selected food and environmental inventories (3) compile characterization factors to assess impacts on biodiversity. Additionally, modifying Agribalyse 3.0 required to also modify the characterization method on Land Competition. This data paper compiles all the data used to obtain the results presented in the companion article entitled: *Environmental trade-offs of fulfilling nutritionally adequacy with reduced animal protein share for French adult populations*[1]*;* i.e. (i) the characterization methods used, (ii) the modifications made to Agribalyse 3.0 and (iii) the nutrient content and quantities consumed of generic foods (iv) the optimized quantities of simulated diets reaching nutrient recommendations with low share of animal-based proteins. It also comprises (iv) Life Cycle Impact Assessment for all Agribalyse 3.0 processes of food having a CIQUAL code (2,497 processes).

**Specifications Table**

**Table:** 

Subject	Environmental engineering
Specific subject area	Environmental assessment of human diets
Type of data	Tables, text CSV, text
How the data were acquired	Average consumption (g/j) and nutritional composition (/100g) of 212 foods products were estimated based on INCA2 data following a previously published method (see details in the following). Optimized quantities were obtained following the methodology described in a previously published paper (see details in the following). Life Cycle Impact Assessment is calculated using SimaPro 9.3 software [2], with native characterization methods, and characterization factors specific to biodiversity and land use derived from scientific literature (see details in the following). A complementary file also provides LCIA results using all indicators of EF 3.0 method [3]. Life Cycle Inventory is derived from Agribalyse 3.0 food database [4], using modified data to be used in the new characterization method. The modifications were made to allow a better classification of land use flows, permitting the assessment of land use impacts on biodiversity without compromising the calculation of other indicators.
Data format	Raw Filtered
Description of data collection	Raw data are issued from open access databases, and scientific literature. They were used in models (diets) and modified to allow calculations (database and characterization methods). Then they provided impact assessment results.
Data source location	Agribalyse 3.0 • Institution: ADEME • City/Town/Region: Nantes • Country: France • https://data.ademe.fr/datasets/agribalyse-synthese CIQUAL • Institution: Agence nationale de sécurité sanitaire de l'alimentation, de l'environnement et du travail • City/Town/Region: Maisons-Alfort • Country: France https://ciqual.anses.fr/#/cms/mentions-legales/node/22 INCA2 • Institution: Agence nationale de sécurité sanitaire de l'alimentation, de l'environnement et du travail • City/Town/Region: Maisons-Alfort • Country: France https://www.anses.fr/fr/system/files/PASER-Ra-INCA2.pdf
Data accessibility	Repository name: LE FEON, SAMUEL; VIEUX, FLORENT; GENESTE, CHRISTOPHE; GAZAN, ROZENN; DARMON, NICOLE; PEYRAUD, JEAN-LOUIS; AUBIN, JOEL, 2022, “Datasets on the nutritional and environmental (including biodiversity) characteristics of food products consumed in France.” Data identification number: https://doi.org/10.57745/HZSRHZ Direct URL to data: https://doi.org/10.57745/HZSRHZ Location: https://entrepot.recherche.data.gouv.fr/

## 1 Value of the Data

- The dataset is an original add-on to Agribalyse as it links nutritional values and environmental impacts including biodiversity.
- They can benefit to everyone who wants to make multicriteria assessment of food products and diets including effects on biodiversity.
- These data are useful as, to our knowledge, currently no published dataset allowing to assess the impact on biodiversity of food products with LCA exist.
- The modification of the Agribalyse 3.0 permits to evaluate biodiversity impacts without compromising results on Land Competition and other indicators.
- Optimized quantities associated to each food provide an example of nutritionally adequate diet with low share of animal proteins.

## 2 Objective

The dataset has been built in support to a research article named: “Reducing the share of animal protein in a nutritionally adequate diet modelled for the French population has mixed environmental consequences” combining different approaches of nutrition and environmental assessment. The extraction and modification of native data from different sources (CIQUAL, Agribalyse, INCA2) permitted to create an original dataset, which have a specific interest for scientists working on the interactions of human nutrition and the environment. Therefore, the objective of the dataset is to give an extensive access to the data supporting the paper, in a perspective of transparency, but also to provide material to the scientific community for further researches.

## 3 Data Description

**Fig. 1:**
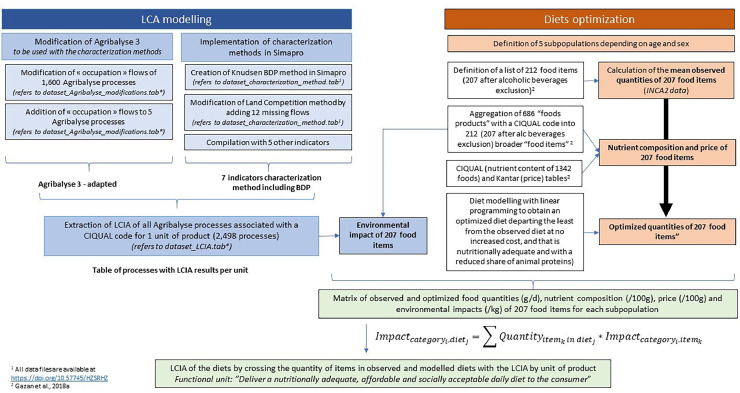
Summary of the methodology used to provide results presented in the companion scientific paper and positioning of the different datasets delivered in this datapaper.

The dataset associated to this article contains files with information on inventory data used for the Life Cycle Assessment (LCA) and the Life Cycle Impact Assessment (LCIA) results presented and discussed in the companion article (Fig. 1). The dataset are not directly usable in LCA software. Considering the frequent evolution of Agribalyse, all the information necessary to modify any version of the database is provided, but not the modified inventory database usable in LCA softwares itself.

1. dataset_Agribalyse_modifications: this dataset describes the modifications made on the name of the Agribalyse 3.0 database instances in order to apply the impacts assessment on biodiversity.
2. dataset_characterization_methods: this dataset describes the characterization factors associated to biodiversity and land occupation flows (from CML non-baseline method, that is a characterization method developed in the university of Leiden) to ensure calculations of Land occupation and biodiversity impact categories.
3. dataset_LCIA: this dataset extracts LCIA results of 2497 food from Agribalyse 3.0 processes using described characterization methods, and their association to nutritional quality databases inputs using characterizations methods assessed in the companion scientific paper.
4. dataset_LCIA_EF_3.0: this dataset extracts LCIA results of 2497 food from Agribalyse 3.0 processes using described characterization methods, and their association to nutritional quality databases inputs using EF 3.0 as characterization methods. EF 3.0 was selected as reference method selected by the European Commission for Product Environmental Footprint calculation.
5. dataset_diets: This database contains average consumption, nutrient composition and environmental impact data of foods of different people categories in France as well as optimized quantities obtained in a previously published study [5].
6. Variable_description: this file provides the description of the variables, with units.
7. Notice: This file explains the content of the dataset and gives some details on methodology.

## 4 Experimental Design, Materials and Methods

### 4.1 Characterization of the impact on biodiversity

Integrating the impacts on biodiversity in agro-food LCA studies is still an emerging issue and several proposals exist, based on different biodiversity levels (genetic diversity, species diversity or ecosystem diversity) or functions [6,7] proposed a method based on the aggregation of three indicators describing the vascular plants’ richness [8], [9], [10]. These indicators consist in associating a characterization factor to a type of land use, translated into an occupation flow (e.g. “occupation, annual crop”) in LCA databases. Our dataset is based on the compilation of characterization factors proposed by [7]. Two characterization factors were added related to – conventional and organic – fruit production [8,11]. Characterization factors used to evaluate the impacts on biodiversity are available in dataset_characterization_methods.

### 4.2 Agribalyse modifications

Agribalyse is the French Life Cycle Inventory database for agricultural and food products [4]. Agribalyse database has not been developed initially with the objective to use the indicator of biodiversity presented in the previous section. Consequently, some processes use occupation flows that are inappropriate to its application, as they do not discriminate agriculture practices, such as conventional and organic productions. For example, the process named “Apple, organic, first production years (phase), at orchard/FR U” uses “Occupation, permanent crop, fruit” as occupation flow. If applying biodiversity indicator previously presented, the same characterization factor will be then associated to conventional and organic fruit productions. Then, it is necessary to modify the occupation flows used in Agribalyse towards flows that are more representative of the occupation and practices in order to discriminate biodiversity performances.

The dataset_Agribalyse_modifications lists all the modifications made to ensure the applicability of biodiversity indicators. They mainly concern processes related to meadows, and organic and tropical productions. The overall modified database can be delivered on demand to the authors.

### 4.3 Modifications of land competition method

Land Competition is a characterization method that consists in calculating the total area used by a system under study. It uses occupation flows and consider the same impact whatever the type of occupation. This means that all characterization factors are equal to 1 m²a / m²a. As some occupation flows are not characterized in the Land Competition method, the modifications of Agribalyse previously presented lead to modify the LCIA results. For example, “Occupation, permanent crop, fruit” was initially characterized in Land Competition method but “Occupation, permanent crop, fruit, organic” is not. Then moving from the first to the second in a process (e.g. “Apple, organic, first production years (phase), at orchard/FR U”) lead to change the results. Then all necessary occupation flows were added to Land Competition method from CML-IA non-baseline v3.04, with a characterization factor equal to 1. Added characterization factors are available in dataset_characterization_methods.

### 4.4 Life cycle impact assessment

Simapro v9.3 software was used to assess the impacts of all processes from Agribalyse associated to a CIQUAL code (https://ciqual.anses.fr/). The assessment was conducted using the biodiversity method previously presented, the modified Land Competition method, as well as 6 other indicators: Climate Change, Acidification, Marine Eutrophication and Freshwater Eutrophication (from EF 3.0 method), Water scarcity (based on AWARE 1.02) and Cumulative Energy Demand (based on CED 1.11). The results are available in dataset_LCIA. The environmental performances of all foods having CIQUAL code from the Agribalyse 3.0 database are presented in this dataset (2,497 processes). A short list corresponding to the data used in the associated article is also presented (676 processes).

### 4.5 Diets

Dataset_diets contains average consumption, nutrient composition and environmental impact data of foods in France. Average consumption was estimated based on INCA2 database, nutrient composition from CIQUAL and environmental from Agribalyse V3. Method of estimation for food consumption and nutrient composition is described in detailed in [12]. Detailed methodology to obtain optimized quantity associated to each food is available in a previously published study [5]. First tab (content) explicit the name of the columns of the data table.

## Ethics Statements

This work did not involve human subjects or laboratory animal, therefore did not meet any ethical issues.

## CRediT authorship contribution statement

**Samuel Le Féon:** Methodology, Investigation, Formal analysis, Writing – original draft. **Florent Vieux:** Conceptualization, Methodology, Formal analysis, Writing – review & editing. **Christophe Geneste:** Software, Investigation. **Rozenn Gazan:** Conceptualization, Methodology. **Nicole Darmon:** Conceptualization, Methodology, Supervision. **Jean-Louis Peyraud:** Supervision, Funding acquisition. **Marion Tharrey:** Methodology, Formal analysis. **Joel Aubin:** Conceptualization, Methodology, Writing – original draft.

## Data Availability

Datasets on the nutritional and environmental (including biodiversity) characteristics of food products consumed in France (Original data) (Dataverse).
